# A single nucleotide variant in the promoter region of the *CCR5* gene increases susceptibility to arthritis encephalitis virus in goats

**DOI:** 10.1186/s12917-019-1979-5

**Published:** 2019-07-06

**Authors:** Silvia Colussi, Rosanna Desiato, Chiara Beltramo, Simone Peletto, Paola Modesto, Maria Grazia Maniaci, Valentina Campia, Antonio Quasso, Sergio Rosati, Luigi Bertolotti, Giuseppe Ru, Pier Luigi Acutis

**Affiliations:** 1 Department of Genetics and Immunobiochemistry, Istituto Zooprofilattico Sperimentale del Piemonte Liguria e Valle d’Aosta, Via Bologna 148, 10154 Torino, Italy; 2 Unit of Biostatistics, Epidemiology and Risk Analysis (BEAR), Istituto Zooprofilattico Sperimentale del Piemonte, Liguria e Valle d’Aosta – Via Bologna 220, 10154 Torino, Italy; 3 Department of Prevention ASL AT, Veterinary Services – Animal Health Unit, Via Conte Verde 125, 14100 Asti, Italy; 4 Department of Veterinary Science, University of Turin, Largo Braccini 2, 10095 Grugliasco (TO), Italy

**Keywords:** *CCR5* gene, Caprine arthritis encephalitis virus, Goats, Marker-assisted selection, Susceptibility

## Abstract

**Background:**

The small ruminant lentiviruses (SRLVs) are a heterogeneous group of viruses that includes caprine arthritis encephalitis virus (CAEV) and Maedi-Visna virus (MVV). SRLVs affect the production and welfare of sheep and goats worldwide. There is currently no effective treatment. Their high mutation rate precludes vaccine development, making innovative control measures necessary.

A variant of the *chemokine (C-C motif) receptor 5 (CCR5)* gene is reportedly involved in resistance to human immunodeficiency (HIV) infection in humans and to SRLV in sheep. The aim of this study was to analyse the genetic structure and variability of the *CCR5* gene in goats and to carry out a cross-sectional study to investigate the role of *CCR5* genetic variants in controlling susceptibility/resistance to CAEV.

**Results:**

The variant g.1059 T located in the promoter region revealed an interesting association with high proviral loads (a 2.8-fold increased risk). A possible explanation could be an alteration of the transcriptional level. Overexpression of the CCR5 receptor on the cell surface may increase virus internalization and proviral load as a consequence.

**Conclusions:**

Our findings could be advantageously used to reduce the susceptibility of goat herds to CAEV by negatively selecting animals carrying the g.1059 T mutation. Eliminating animals predisposed to high proviral loads could also limit the development of clinical signs and the spread of the virus, since these animals are also highly efficient in shedding the virus.

**Electronic supplementary material:**

The online version of this article (10.1186/s12917-019-1979-5) contains supplementary material, which is available to authorized users.

## Background

The small ruminant lentiviruses (SRLVs) are a heterogeneous group of viruses that includes caprine arthritis encephalitis virus (CAEV) and Maedi-Visna virus (MVV). They are enveloped RNA viruses belonging to the lentivirus genus of the *Retroviridae* family, which also includes the human immunodeficiency virus (HIV). SRLVs infect monocytes and macrophages and cause persistent infections and chronic debilitating diseases in sheep and goats [1]: 20–30% of infected goats exhibit clinical manifestations characterized by emaciation, progressive arthritis, and mastitis [2].

SRLV affects the production and welfare of sheep and goats worldwide; the estimated seroprevalence is 80–90% in dairy goats [3, 4] and 60–82% in Italy [5, 6]. There is no effective treatment for SRLV infection; the virus’s high mutation rate precludes vaccine development. Herd management is commonly used to prevent viral transmission mainly by culling infected animals based on serology. However, variation in serological response within a single animal and at the flock level hampers serological diagnosis [7]. In herds with high seroprevalence, an alternative approach is the selection of negative, artificially fed progeny [8]. But because this is both expensive and time consuming, innovative control measures are urgently needed.

A variant in the *chemokine (C-C motif) receptor 5 (CCR5)* gene has been reportedly involved in resistance to HIV infection in humans and to SRLV in sheep [9, 10]. Together with CD4, he human chemokine receptor CCR5 serves as the principal co-receptor for macrophage-trophic (R5) HIV strains. Numerous genetic variants in the coding or the promoter region have been identified in various ethnic groups. Caucasians with the coding deletion delta-32 have no functional CCR5 protein on the cell surface and are highly resistant to HIV infection [9]. Similarly in sheep, a common 4-base deletion in the promoter region of *CCR5* has been reported to reduce the proviral level, with a 3.9-fold differential transcription in heterozygous animals [10].

The aim of this study was to analyse the genetic structure and variability of the *CCR5* gene in goats and to carry out a cross-sectional study to investigate the role of the genetic variants in controlling susceptibility/resistance to caprine arthritis encephalitis virus for future use in marker-assisted selection breeding schemes.

## Results

The caprine *CCR5* sequence showed two exons with the entire open reading frame (ORF) located on exon II, as described for the ovine *CCR5* gene. The CDS encodes a 352 amino-acid protein with 98% of similarity compared with the ovine sequence. Promoter region and complete CDS of the caprine *CCR5* gene was deposited in GenBank (Accession number HQ650162).

Table 1 presents the variants and relative frequencies found in the survey. All the variants respected Hardy-Weinberg assumption; no 4-base deletion at the promoter level was found as is instead described for sheep.

**Table 1:** *CCR5* variants found in the survey, position and frequencies (Caprine Reference Sequence HQ650162.1)

Variation	Region of interest	Frequency % (MAF)
g.186_188InsTC	Promoter	0.17
g.226 G > A	Promoter	0.17
g.551 C > G	Promoter	0.14
g.925 C > A	Promoter	0.17
g.1059 C > T	Promoter	0.19
g.1394 C > T	Promoter	0.17
g.2170 T > C	Intron	0.17
g.2573 A > C	Intron	0.03
g.2791 C > T	Intron	0.14
g.3023 T > C	Intron	0.03
g.3167 T > C	Intron	0.03
g.3203 T > C	Intron	0.17
g.3876 T > C	Exon 2 CDS	0.14
g.4553 C > T	Exon 2 3′-UTR	0.17
g.4918 C > T	Downstream 3′- UTR	0.14
g.5089 G > C	Downstream 3′- UTR	0.03
g.5110 G > C	Downstream 3′- UTR	0.03

Twenty-two cases (5 wild type, 17 mutated) and 68 non-cases (39 wild type and 29 mutated) were analysed. SNP g.1059 T was missing in one goat and the sample was removed from the association study. The chi-square test demonstrated the presence of eight SNPs statistically associated with high proviral load (Table 2). Subsequent correction for multiple testing resulted in a loss of statistical significance for each SNP, as Bonferroni and Sidak corrections assume each SNP to be independent of each other and fail to take linkage disequilibrium into account. Hence, many SNPs were in close linkage disequilibrium to one another and a correlation between SNP alleles was detected. A permutation test was performed and empirical *p*-values were determined through 10,000 permutations. The results showed that the g.1059 T mutation was significantly associated with high proviral load, also after correction for multiple testing, with a higher proviral load in the individuals carrying the g.1059 T mutation. Various alternatives of setting the cut-off led to similar results, pointing to an association between SNP 1059 and high proviral load values. The g.1059 T mutation was entered as an independent variable into a univariate analysis based on the mixed-effects Poisson regression model: the prevalence ratio (PR) considering the crude effect of the SNP was 3.25 (95% confidence interval [CI] 1.2–8.81, *p* = 0.020) in the goats carrying the g.1059 T mutation.

**Table 2:** *CCR5* variants associated with the proviral load

Variation	CHISQ	*P*-value
g.1059 C > T	6.092	0.01358
g.4353 C > T	5.558	0.01839
g.925 C > A	5.077	0.02425
g.2170 T > C	4.623	0.03154
g.3203 T > C	4.623	0.03154
g.226 G > A	4.62	0.03161
g. 186_188InsTC	4.175	0.04102
g.1394 C > T	3.907	0.04809

Chi square test results (CHISQ) and *P* value < 0.05 (*P*)

After adjusting for animal age, a 2.8-fold increase in risk was still evident for the animals carrying the g.1059 mutation (C/T and T/T) versus wild type (PR 2.81; 95% CI 1.01–7.837; *p* = 0.047) (Table 3). No significant association was found with increasing age (*p* = 0.081) or with any interaction terms. Haplotypes analysis identified 27 haplotypes and none of them was associated to proviral load (Chi-square test). Moreover a permutation test was performed and a non significant result was obtained (*p* = 0.25).

**Table 3:** Results of a Poisson mixed-effects regression model

Mixed-effects Poisson regression model	Genotype	PR	p-value	[95% CI]
g.1059 T	C/T	2.82	0.047	1.01–7.83
Age in months		1.03	0.081	1.00–1.06
_cons		0.03	0.000	0.00–0.22

Genotype and age (aa a continuous variable in months) were entered in the model as covariates to evaluate the potential association with proviral load

The bioinformatic analysis did not predict with high probability any transcription factor binding site related to the SNP g.1059. Nevertheless, EMSA was carried out to experimentally assess the presence of binding sites using synthetic oligos carrying the two SNP g.1059 alleles and the nuclear extract from the caprine buffy coat. An unspecific shift was visualized, not silenced by the corresponding unlabelled ds oligo, as competitor (See Additional file 1). The EMSA experiments indicated that no binding site for transcription factor from buffy coat is present at the SNP g.1059 locus.

## Discussion

Caprine *CCR5* gene structure and variability is described here for the first time. The variant g.1059 T, located in the promoter region, revealed an interesting association with high proviral loads (a 2.8-fold increased risk). A possible explanation for this is alteration of the transcriptional level, specifically, overexpression of the CCR5 receptor on the cell surface. This could increase virus internalization and proviral load as a consequence. A further explanation would be that the variant g.1059 T might not be directly associated with the high proviral load, but rather that it is in linkage with another unknown functional variant outside the present dataset.

We found no interaction between the nuclear extract from the goat buffy coat and the DNA region where the SNP is located; this suggests that the SNP did not alter the transcription factor (TF)F sites. Studies using transient cell-based promoter assays have shown, however, that one-third of SNPs located within the 500 bp region upstream from the transcriptional start site (TSS), like g.1059 T, may affect transcription levels by 50% or more; sequence − 300 to − 50 bp from the TSS are known to positively contribute to core promoter activity [11].

## Conclusions

Our findings could be advantageously used to reduce the susceptibility of goat herds to CAEV by negatively selecting animals carrying the g.1059 T mutation. Eliminating animals predisposed to high proviral loads could also limit the development of clinical signs and the spread of the virus, since these animals are also highly efficient in shedding the virus [7]. The implementation of marker-assisted selection schemes could offer a complementary, innovative strategy to control CAEV infection, especially in herds with high seroprevalence levels.

## Methods

### Study of the *CCR5* structure in goats

DNA was extracted using the kit Pure Link™ Genomic DNA Mini Kit (Invitrogen) from the blood of four Chamois coloured goats. PCRs were carried out to cover the putative promoter region, Exon I, Intron 1, Exon II, and the 3′-UTR region of the *CCR5* gene according to the protocol and the primers described by White et al. [10]. PCRs were carried out in a total volume of 50 μl using Platinum® qPCR Supermix-UDG (2X) (Invitrogen) and containing 50–100 ng of genomic DNA and 300 nM of each primer. Amplicon dimensions were visualized on 2% agarose gel under UV light (Gel Doc 2000, Bio-Rad). Products were labelled using BigDye® v3.1 chemistries (Applied Biosystems, Thermo Fisher Scientific). Sequencing primers were reported by White et al. [10]. Capillary electrophoresis was done on an ABI3130 genetic analyser (Applied Biosystems, Thermo Fisher Scientific).

MegAlign™ software (DNASTAR) was used to align the *CCR5* ovine reference sequence (GenBank accession number FJ008056) and the caprine sequences obtained and not previously reported. The *CCR5* caprine sequence was first gathered by homology with the ovine one and then experimentally verified through c-DNA amplification. The free on-line software Primer3 was used for designing the two primers: EXIF 5′-CCAACTCAGAAGAAACTGCAT-3′ (1427–1447 ovine reference sequence) and EXIIR 5′-AAGCAAACACAGCATGAACG-3′ (3692–3673 ovine reference sequence). PCR was carried out in a total volume of 25 μl. Table 4 lists the reagents and relative volumes. PCR conditions were: 95 °C for 10 min, followed by 35 cycles at 94 °C for 30 s, 54 °C for 30 s, 72 °C for 60 s, and a final extension at 72 °C for 7 min. Amplicon length (533 bp) was visualized on 2% agarose gel under UV light (Gel Doc 2000, Bio-Rad). Products were labelled using BigDye® 3.1 chemistries (Applied Biosystems, Thermo Fisher Scientific). PCR primers were also used for full amplicon sequencing. Capillary electrophoresis was done on an ABI3130 genetic analyser (Applied Biosystems, Thermo Fisher Scientific).

**Table 4:** Reagents used for PCR for defining Caprine *CCR5* structure

Buffer 10X	2.5 μl
Mg ^2+^ (2.5 mM)	2.5 μl
DNPTs (0.2 mM)	1.0 μl
Primer ExIF (300 nM)	0.5 μl
Primer ExIIR (300 nM)	0.5 μl
GC solution	5 μl
Taq Roche(1.5 U)	0.2 μl
Water	11.8 μl
c-DNA (dil. 1/5)	1 μl

### Survey of chamois goats and functional analysis of the SNPs

A genetic survey of the *CCR5* gene was carried out on 20 Chamois coloured, geographically unrelated goats (sex-ratio 1:1). The methodology was reported above in “Study of the *CCR5* structure in goats”. Lasergene SeqMan software (DNAStar) was used for sequence alignment. Sequences derived from goats sampled for the case-control study were aligned using the caprine reference sequence deposited by our group in GenBank (Accession number HQ650162).For all SNPs found in the survey, freely available on-line software was used to predict the role of each SNP allele in creating/disrupting putative TFBS in regulatory regions. In particular, each SNP was analysed in parallel with TFBIND, ALGGEN-PROMO, MATCH and JASPAR software in order to evaluate agreement of predictions by different algorithms and to minimize false positives.

### Cross-sectional study

Ninety one Chamois coloured goats were recruited from two herds. A cross-sectional study was carried out in order to identify polymorphisms and mutations in the goat herds where the CAEV was circulating and to investigate its role in determining susceptibility and/or resistance to the disease

Real-time PCRs were used to quantify the proviral load for each animal in the study. Peripheral blood mononuclear cells (PBMCs) were isolated by Ficoll gradient centrifugation (1.077) from EDTA-treated blood. DNA was extracted from PBMCs using a DNA Blood Mini Kit (Qiagen). Proviral load was estimated by quantitative real time PCR as previously described [7]. Ten-fold serial dilutions ranging from 10^6^ to 1 copies of a plasmid (pDRIVE, Qiagen) containing the gag region were used to generate a standard curve and compared to each sample. The results are expressed as provirus copy number/50 ng extracted DNA.

Because all the animals were potentially infected though asymptomatic, the level of infection within the herd (proviral load) was modelled as a dependent variable (infection prevalence) in the cross-sectional study and used to define cases and controls. Actually, proviral load has been reported to be essential in early diagnosis of the infection, also in absence of clinical signs [8]. Moreover SRLV was easily isolated from high proviral load animals whereas low proviral load animals did not shed virus [7]. Proviral load was categorized based on different percentile values: the 25th percentile, 50th percentile, the parametric method on log-normal distribution and on quartile. Hence, case definition was based on the individual proviral load: animals with a proviral load greater than or equal to the 75th percentile of the data distribution (456.43 copy number/50 ng DNA) were classified as cases and the others as non-cases.

Data quality control and statistical analysis were performed in accordance with Anderson [12]: removal of individuals with missing genotyping or SNPs not genotyped, evaluation of Hardy-Weinberg equilibrium, and minor allelic frequency (MAF). SNPs genotyped in less than 95% of samples, SNPs with MAF < 0.1%, and samples that did not respect the assumption of Hardy-Weinberg equilibrium were removed.

The first step in the genetic association study was to assess the association between allelic variants and proviral load for each SNP according to Pearson’s chi-squared and Fisher’s exact test. Among the SNPs associated with a high proviral load, the presence of linkage disequilibrium was studied and adjusted by permutation correction for multiple testing and collinearity. SNPs significantly associated with the disease (*P* < 0.05) based on the chi-square test were used as candidates for further analysis. A mixed-effects Poisson regression model was fitted with individual SNPs entered as an independent variable, age (in months) as a potential confounder, and the herd as random effect to take into account the grouping structure of the data. Age was included in the model as it was statistically associated with proviral load at univariate analysis; the potential for statistical interaction between age and polymorphisms was also tested. Data quality control and association tests were performed using PLINK software (v 1.07) [13]; data analysis and model fitting were carried out using Stata Statistical Software, release 14.0 (Stata Corp.) [14]. Haplotype analysis and permutation test were carried out, using Phase software v.2.1.1. [15].

### Electrophoresis mobility shift assay (EMSA)

NE-PER Nuclear and Cytoplasmic Extraction Reagents (Thermo Scientific) with Halt Protease Inhibitor Single-use Cocktail EDTA-free (Thermo Scientific) were used to extract the nuclear fraction from the buffy-coat (leucocytes) of anticoagulated goat blood; nuclear extracts were quantified by fluorometric quantification. The regions of the *CCR5* gene, including the SNPs of interest, were used as a template to design double-strand (ds) DNA oligos for the EMSA (Table 5). 3′ biotinylated and non-biotinylated DNA ds oligos were synthesised for both SNP alleles as a single strand. Annealing was performed as previously described by Holden and Tacon (2011) [16]. EMSA was performed following the manufacturer’s instructions for the LightShift® Chemiluminescent EMSA Kit (Thermo Scientific), using DNA retardation gels 6% (Thermo Scientific) and Biodyne B pre-cut membranes. The Chemiluminescent Nucleic Acid Detection Module (Thermo Scientific) was used to detect chemiluminescent signals on X-ray film.

**Table 5:** Sequences and melting temperature (Tm) of the oligos for EMSA

Oligo name	Sequence 5′-3’	Tm
CCR5_SNPg.1059_C_f	CTGGCTTCCACAAA**C**ACCAAGTGGGTCAGA	76 °C
CCR5_SNPg.1059_C_r	TCTGACCCACTTGGT**G**TTTGTGGAAGCCAG	
CCR5_SNPg.1059_T_f	CTGGCTTCCACAAA**T**ACCAAGTGGGTCAGA	73.3 °C
CCR5_SNPg.1059_T_r	TCTGACCCACTTGGT**A**TTTGTGGAAGCCAG	

The polymorphic nucleotides of the SNPg.1059 are given in bold

## Additional file

Additional file 1: EMSA gel using double-strand oligo with SNP g.1059 (B) and nuclear extract (NE) from goat buffy coat. (DOC 394 kb)

## Data Availability

The *CCR5* caprine reference sequence generated during the current study and the *CCR5* ovine reference sequence used for the alignment are available in the GenBank freely available repository (https://www.ncbi.nlm.nih.gov/genbank/), accession number HQ650162.

## Abbreviations

CAEV: caprine arthritis encephalitis virus
*CCR5*: *chemokine (C-C motif) receptor 5* gene
CDS: DNA coding sequence
EMSA: electrophoresis mobility shift assay
HIV: human immunodeficiency virus
MVV: Maedi-Visna virus
SNP: single nucleotide polymorphism
SRLV: small ruminant lentiviruses
TSS: transcriptional start site
UTR: untranslated region
