# Supplementary figures and images for: A single nucleotide variant in the promoter region of the CCR5 gene increases susceptibility to arthritis encephalitis virus in goats
Source: BMC Vet Res. 2019 Jul 6;15:230. doi: 10.1186/s12917-019-1979-5 (PMC6612200; doi:10.1186/s12917-019-1979-5)

**
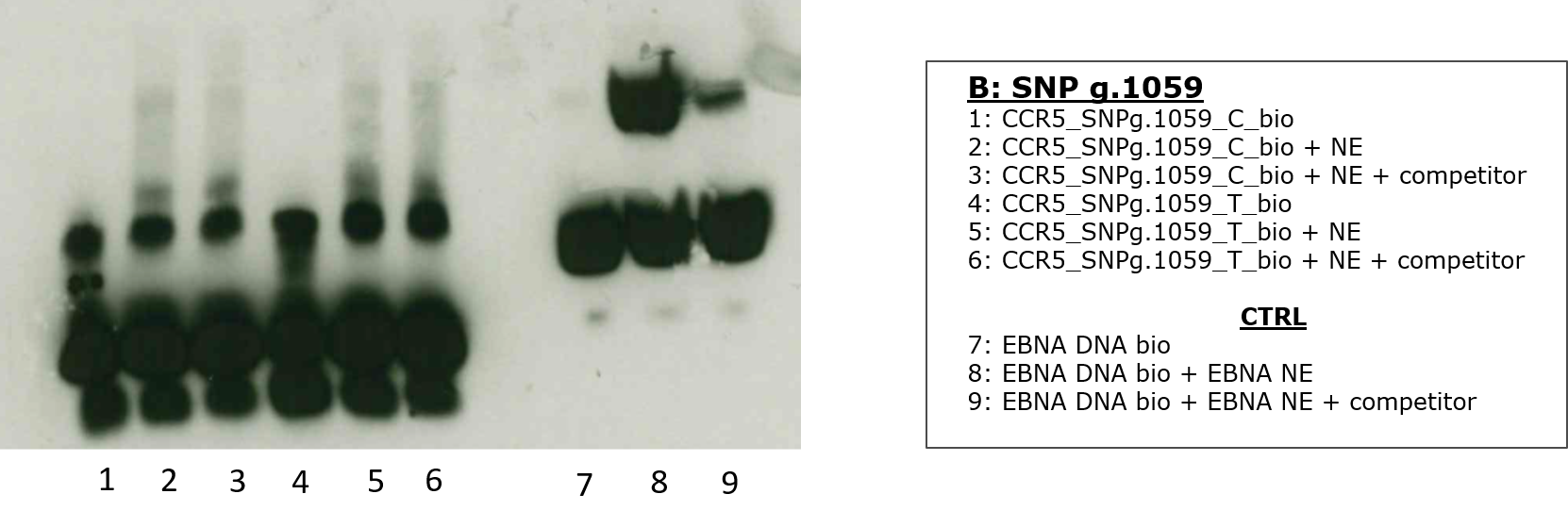
**

Supplement: Supplementary file 1 — EMSA gel using double-strand oligo with SNP g.1059 (B) and nuclear extract (NE) from goat buffy coat. (DOC 394 kb) [file 12917_2019_1979_MOESM1_ESM.doc]
